# The Relationship between Fear of Infection and Insomnia among Dentists from Oradea Metropolitan Area during the Outbreak of Sars-CoV-2 Pandemic

**DOI:** 10.3390/jcm10112494

**Published:** 2021-06-04

**Authors:** Magdalena Iorga, Raluca Iurcov, Lavinia-Maria Pop

**Affiliations:** 1 Behavioral Sciences Department, “Grigore T. Popa” University of Medicine and Pharmacy, 700115 Iasi, Romania; 2 Faculty of Psychology and Education Sciences, “Alexandru Ioan Cuza” University, 700111 Iasi, Romania; lavinia.pop@student.uaic.ro; 3 Dentistry Department, Faculty of Medicine, University of Oradea, 410073 Oradea, Romania

**Keywords:** pandemic, dentist, stress, anxiety, fear, insomnia, mental health, preventive behavior, fear of Covid-19, Athens Insomnia Scale

## Abstract

Various studies have shown the impact of COVID-19 pandemic on mental health, identifying that people with a strong fear of getting infected are more prone to become stressed, depressed, anxious and to experience sleeping disturbance. The present study focuses on the impact of fear of COVID-19 and its relationship with insomnia among dentists. 83 dentists from public and private clinics were included in the research. A questionnaire was especially constructed for this study, consisting of three parts: the first part gathered socio-demographic and medical data, and a succession of self-rated items collected opinions about lockdown and preventive behaviors; the second part evaluated the level of fear of infection with Coronavirus-19 using the Fear of Covid 19 Scale; the third part investigated the presence of insomnia using the Athens Insomnia Scale. Collected data were processed using SPSS (v. 25). The total scores for fear of COVID 19 and insomnia were assessed. A strong positive correlation was identified between the total score of AIS and the total score of FCV-19S. The fear of COVID-19 had a significant positive correlation with the practice of several preventive behaviors. Dentists with chronic diseases were found to be more prone to suffer from insomnia than healthy dentists. Significant differences between women and men in terms of night symptoms were discussed. The findings are useful for dentists and policy makers to evaluate the impact of fear of infection on mental health status.

## 1. Introduction

Since March 2020, when the World Health Organization (WHO) declared infection with COVID-19 as a pandemic [1] dentists have had to deal with many challenges: the COVID-19 pandemic has influenced the physical and mental health of dentists and impacted their family relationships and financial status, and has led to changes in professional practice [2,3,4,5].

From the beginning of the COVID-19 pandemic, doctors have been considered a hotspot for infection, with a high risk of bidirectional transmission—from doctor to patient and vice versa. The risk of contagion was extremely high, especially in the context of dental medical services: blood droplets and saliva that are deposited on the surfaces or aerosol inhalation generated by rotating instruments and ultrasound hand pieces put both doctors and patients at high risk [2,3].

Tysiąc-Miśta and Dziedzic [4] showed that one of the most important challenges for Polish dentists was that of medical protective equipment. Their study pointed out that 71.2% of dentists decided to suspend their clinical practice during the onset of the pandemic. Dentists who continued their clinical work during the first months of the pandemic were four and a half times more likely to have access to PPE than those who closed their dental offices. The same results were highlighted by Chamorro-Petronacci et al. [5] among Spanish dentists. The authors found that only 12.3% of participants could obtain personal protective equipment (PPE) including FFP2 masks, and dentists registered important economic losses due to the interruption of their activity. Casillas Santana et al. [6] showed that only a small percentage of Mexican dentists used protective masks and a large majority had to change disinfection rules between appointments to ensure a safe environment for patients. Consolo et al. [7] found that dentists from northern Italy reported feelings of concern (70.2%), anxiety (46.4%) and fear (42.4%) and most of them (89.6%) mentioned increased concerns about their professional future. Ahmed et al. [8] conducted a survey among dentists from thirty countries and found that 90% were anxious while treating a coughing patient or a patient suspected of being infected with COVID-19. More than 72% of the participants felt nervous when talking to patients in close proximity, and most of them (92%) were afraid of carrying the virus from workplace to their families. The authors also found that 73% were anxious about the cost of the treatment if they got infected, and 86% about the mortality rates presented by the media. More than half of the dentists recommended the closure of private and public dental clinics until the number of COVID-19 cases started to decline [8].

Shacham et al. [9] conducted a cross-sectional survey among Israeli dentists and dental hygienists and found higher psychological distress among those with history of illness, with an increased level of fear of contracting COVID-19 from their patients, and with those who self-rated their work as overloaded. Vergara-Buenaventura et al. [10] showed that there were important health consequences of the coronavirus disease 2019 pandemic in dentistry, including physical and psychological pressure, depression, social anxiety, and other mental health concerns. Sarialioglu Gungor et al. [11] highlighted that Turkish dentists had to deal with a high risk of infection, isolation from their families, discrimination, and overwork with inadequate protection, developing frustration and exhaustion. In Egypt, Hanafy identified that 75.1% of dentists reported difficulties in finding Personal Protective Equipment and 97.6% of the questioned doctors reported worry about acquiring COVID-19 while working. When questioned about it, 44% of participants reported anxiety, 28.7% concern, and 16.4% fear [12]. In a study conducted by Moraes et al. [13] of Brazilian dentists it was shown that there were three major impacts of the pandemic on dentistry: increasing inequalities due to health coverage differences between public and private clinics, the adoption of new clinical daily practices (correlated with an economic burden for dentists), and associations of regional COVID-19 incidence/mortality with fear of contracting the disease in the working environment. All these studies revealed that the Coronavirus-19 pandemic has had, since the beginning, a great impact on dentists’ personal and professional lives, with important psychological consequences. Fear, anxiety and stress should be further investigated, due to their consequences regarding general health status.

Identifying the level of fear has a dual scientific purpose. The positive aspect of fear is that it is an alarm signal; fear is a normal reaction and determines awareness about a situation, produces changes in behaviors, and stimulates adaptability [9]. In the early stages of previous pandemics, such as Ebola or MERS, fear was associated with increased vaccination rates and the practice of preventive behaviors like hand washing, distance, body hygiene, etc. [14]. The negative side of fear is that the presence of high scores for fear is associated with the appearance and persistence of psychological problems such as anxiety, depression, post-traumatic stress disorder, sleeping disorder, eating disorder, or somatization [9,10,14,15,16]. Authors have shown that a fear construct is expected to have a significant predictive power of posttraumatic stress symptomatology, anxiety, and insomnia, since the most common clinical manifestations of fear include these psychological problems [15]. Therefore, the presence of fear determines the sufferer to search for preventive behaviors but also has negative consequences on physical and mental health [8,14]. That is why, when analyzing fear in the context of COVID-19 pandemic, as Nikopoulou et al. highlighted in their study [16], it is extremely important to discriminate between adults with extreme COVID-19-related fear and those with normal fear reactions, to prevent psychological consequences and to maintain mental health. In the context of the coronavirus-19 pandemic, the impact on mental health is just at the beginning. Studies have identified the influence of the pandemic on the psychological, physical, social, and financial status as an acute reaction [6,8,14].

Compared to other countries of the European Community, Romania registered a lower risk of community transmission and a considerably lower number of deaths. The first case of infection was identified on 26 February 2020. By Presidential Decree, on 16 March a state of alarm was imposed; on 21 March, by Military Decree, a lockdown was imposed till 15 May [17,18]. Dental offices had to close and only emergency dental offices from public institutions (hospitals or clinics) continued to provide dental services. Due to the small number of emergency dental care offices (less than 5%) private dental clinics could accept patients in compliance with two rules: to provide only a limited amount of dental care (emergency cases) and to have the approval of Territorial Health Departments (after checking that the clinics had respected all the preventive measures to limit the spread of the infection). Only a very small number of private clinics could follow the imposed rules, first because of the difficulty in finding protective suits or protective materials and, second, because of their costs that negatively impacted their financial balance. The third important reason is not to be ignored, that at the beginning of the pandemic, information on the ways of transmitting the virus was relatively limited, which determined both patients and dentists being careful in carrying out activities related to dental services.

A small number of articles were published during the first year of the pandemic about the practice of dental services in Romania and how the restrictions, fear of infection and the new adopted preventives rules impacted dental services. The study of Pituru et al. [19] conducted among Romanian dentists identified that 87.1% of the respondents preferred a full set of personal protective equipment, including gloves, goggles, N95 mask, face shield, head cover, gown and shoe cover while treating asymptomatic patients with positive travel/contact history. Moreover, Petrescu et al. [20] showed that certain pathologies provided in emergency dental rooms in Romania considerably increased during 2020, compared to 2019. In April 2019, 160 patients had visited the Emergency Department of County General Hospital whereas in April 2020, one month after the State of Alarm, 2131 patients sought dental treatment in this department, as other dental emergency centers and private practices were closed by the State of Emergency dispositions. The number of patients increased more than 13 times. The main causes of attendance at emergency dental offices were acute apical periodontitis and acute pulpitis. However, no studies evaluated the impact of the COVID-19 pandemic on the mental health of Romanian dentists.

The goal of the present research is to evaluate the mental status of dentists after the first wave of the COVID-19 pandemic (November–December 2020) by identifying the presence of fear of Covid-19 and insomnia, and their relationship with dentists’ preventive behaviors. As secondary purpose, we wanted to highlight the correlation analysis between fear and the practice of preventive behaviors (as a health-protective consequence of fear) and fear and insomnia (as a negative consequence on health status) among dentists during the first months of the pandemic. This study is part of a larger investigation among dentists; previous results have evaluated preventive behaviors and new preventive practices in dental clinics, adopted by dentists to diminish the risk of infection from and to their patients, and the impact of the pandemic on their family life and financial status [21].

## 2. Materials and Methods

This cross-sectional study was conducted in Oradea department situated in the northwestern part of the country, one of the regions that immediately applied restrictive rules even before the imposed State of Alarm, decided by Presidential Ordinance on 15 March 2020, especially due to its closeness to the Romanian border. The data for the present research was collected between 18 November and 5 December 2020, after the first wave of the pandemic, thus covering the first seven months of the pandemic.

### 2.1. Participants and Data Collection

The questionnaire was created and distributed with the Google Forms application (Alphabet, Mountain View, CA, USA). The survey was distributed online to dentists registered in the College of Dentists of Oradea. The respondents were informed about the purpose of the research and the use of the collected data. The continuation of the questionnaire was assumed as an agreement to take part to the survey. No incentives were offered to the voluntary respondents. The inclusion criteria were dentists registered at that time by the Romanian College of Dentists, Oradea Department, working in public or private dental clinics or Dental Emergency Units. The exclusion criteria were retired or unemployed dentists, incomplete questionnaires (not fully filled in) and questionnaires returned after the deadline. The survey was sent to 125 dentists who previously agreed to receive news or questionnaires; 93 of them filled in the surveys (response rate was 74.4%). We excluded ten questionnaires because the respondents were retired dentists. Finally, 83 forms were included in our databases and considered for the research. In November 2020, 457 dentists were registered at the Oradea College of Dentists (metropolitan area and other cities in the department) so the sample is relevant for dentists working in the region.

### 2.2. Instruments

A questionnaire was used to collect the data for the present research, and contained three parts:

- The first part collected (a) sociodemographic data such as age, gender, length of employment, number of children, marital status, length of employment, environment, type of institutions, level of specialization; (b) medical-related data such as the presence of a chronic disease or having a previous positive diagnosis of infection with the virus; (c) preventive behaviors adopted to diminish the risk of infection with answers rated on a 5-point Likert as following: 1 (never), 2 (rarely), 3 (sometimes), 4 (often) and 5 (always).
- The second part evaluated the fear of COVID-19. The psychological tool used for the present research was Fear of COVID-19 (FCV-19S) elaborated in 2020 by Ahorsu et al. [22]. The tool included seven items, and the respondents had to indicate their level of agreement with the statements using a five-item Likert-type scale. Answers included strongly disagree, disagree, neither agree nor disagree, agree, and strongly agree. The minimum score possible for each question was 1, and the maximum was 5. A total score was calculated by adding up each item score (ranging from 7 to 35). The higher the score, the greater the fear of Cororonavirus-19. The instrument was used worldwide in the first year of the pandemic. Chi et al. [23] (2021) also identified two factors: one was fear thoughts including items 1, 2, 4, and 5, i.e., subjective experience of fear. The other was physical response including items 3, 6, and 7, which represents physiological response [23]. The scale proved its good validity and reliability as psychometric characteristics and versions in many languages and countries were employed, including a Romanian version [16,22,24,25,26,27,28,29,30,31,32,33,34]. The cut-off score was established at 16.5 points
- The third part identified the presence of insomnia using the Athens Insomnia Scale (AIS) of Soldatos et al. [24]. The scale is an eight-item instrument that was designed for quantifying sleep difficulty based on the ICD-10 criteria. It consists of eight items: the first five pertain to sleep induction, awakening during the night, final awakening, total sleep duration, and sleep quality; while the last three refer to well-being, functioning capacity, and sleepiness during the day. The first five address the participant’s night-time symptoms and the other three investigate the daytime impact due to any reported sleep disturbances. Each item was rated on an ordinal scale of 0 (no problem at all) to 3 (very serious problem). The dentists were required to rate the items if they had experienced any sleep difficulty described in each question at least three times a week during the previous month. A maximum total score of 24 indicated the most severe symptoms of insomnia. For this scale, the cut-off point of ≥6 represented a minimum criterion for the confirmation of insomnia symptoms in our respondents.

### 2.3. Statistical Analysis

All analyses were performed using the IBM Statistical Package for Social Sciences (SPSS) Statistics for Windows, version 25(SPSS Inc., Chicago, IL, USA). The descriptive statistics of socio-demographic and medical data were expressed as means and standard deviations (SD), frequencies and percentages. Given that the distribution of data is not normal, nonparametric tests were used. The Mann Whitney test was performed for the comparative analysis among genders and professional categories, as well as for marital and parenthood status. The correlation analysis was done using Spearman correlations. The level of statistical significance was set at *p* < 0.05.

### 2.4. Ethical Approval

The present study was conducted in accordance with the Declaration of Helsinki, and the protocol was approved by the Ethical Committee of the Emergency County Hospital of Oradea, Romania, with the registration number No. 26214/18.11.2020.

## 3. Results

### 3.1. Sociodemographic and Medical Data

Sociodemographic and medical data were gathered, together with professional environment characteristics and family related information. Detailed information is presented in Table 1.

### 3.2. Opinions of Dentists on Infection and Preventive Measures

A succession of items identified the dentists’ opinion about preventive measures such as establishing shorter dental interventions. Two items investigated the respondents’ opinions about the risk of infection in dental settings. Additionally, some items self-rated the fear of infection related to patients (they did not declare the truth about their health; they could transmit the infection to dentists; they did not wear medical masks during dental interventions which increased the risk of contagion) and related to preventive practices (fear of infection due to inappropriate use; the medical suit did not offer 100% protection).

Finally, dentists were asked to rate their state of anxiety when watching the news and reading stories related to COVID-19 pandemic. The answers were rated on a Likert-like scale from 1 (never) to 5 (always) and the results are presented in Table 2.

### 3.3. Fear of COVID-19 Scale (FCV-19S)

For the present research, for the Fear of COVID-19 scale, Cronbach Alpha was 0.89, which proved a good internal consistency.

The total score was 14.56 ± 6.90 (ranging from 7 to 35). No significant differences were identified considering gender (male/female—*p* = 0.311), marital status (single/in relationship—*p* = 0.613), diagnostic of a chronic disease (yes/no—*p* = 0.066), or setting of work (public/private—*p* = 0.196).

The analysis of data showed positive correlations between FCV-19S score and the practice of some preventive behaviors such as shortening the duration of dental consult (r = 0.488 **, *p* < 0.001). This means that participants who were more fearful of Covid-19 were more likely to engage in preventative behaviors, such as shortening the duration of dental consultations.

Strong correlation was also identified related to total score for fear of infection and dentists’ opinions about the fact that dental procedures were a major source of infection (r = 0.415 **, *p* < 0.001), and dentists had an increased risk of infection from the new virus in their patients (r = 0.598 **, *p* < 0.001). Fear of getting the infection from colleagues (r = 0.452 **, *p* < 0.001), fear of infection while taking off the protective suit (r = 0.635 **, *p* < 0.001), and fear of infection since patients cannot wear a mask during a medical treatment (r = 0.370 **, *p* = 0.001) were also found positively correlate with total score for FCV-19S. We must notice here that using self-rated items, we identified a high level of fear of infection from others (dental procedures, colleagues, patients, protective suits) and not the fear of transmitting the infection to others. This aspect will be detailed in the Discussion section.

We found that the number of children influenced the level of fear of infection; the more children the dentists had (r = −0.301 **, *p* = 0.007), the less intense the fear of COVID-19.

A previous study presented that Fear measured with FCV-19S had a bi-factor structure that indicated two dimensions: (1) the first was explained by general fear which reflects cognitive fear or a subjective experience of fear and (2) the second was explained by physical response group factors which indicates somatic fear, the physiological response of the organism [23]. Considering this, the comparative analysis showed that dentists who were university teachers (Mdn1 = 4) scored higher on physical response (Fear of COVID-19 subscale) (z = −2.033, *p* = 0.042) due to having a more intense physical experience, as opposed to those who did not carry out teaching activities (Mdn2 = 3).

### 3.4. Athens Insomnia Scale (AIS)

For our study, the Cronbach’s Alpha for the total group was 0.87. A total score equal or greater than 6 was considered as indicative of insomnia for AIS. Our analysis of data showed a total score of 11.37 ± 3.45, meaning that our respondents showed a high level on this scale. Results ranged from a minimum score of 8 (19.3%, *n* = 16) to a maximum of 24 points (1.2%, *n* = 1). Detailed results are presented in Table 3.

The comparative analysis showed that dentists suffering from chronic diseases (Mdn = 17) had insomnia (z = −2.892, *p* = 0.004) more often than healthy dentists (Mdn = 10). Results of the Mann Whitney test (z = −3.583, *p* < 0.001) showed that dentists with a higher level of fear (Mdn = 14) had a higher score on the questionnaire assessing the severity of insomnia than the dentists who had lower levels for FCV-19S (Mdn = 10). Dentists with a higher level of fear (Mdn = 6) had had fewer working weeks since the outbreak of the pandemic compared (z = −2.900, *p* = 0.004) to dentists with a lower level of fear (Mdn = 9).

There were significant gender differences in terms of symptoms during the night, namely sleep induction, sleep quality and total sleep duration (z = −2.090, *p* = 0.037) in the sense that men (Mdn = 4) scored higher at this subscale and had more problems with night-time symptoms, as opposed to women (Mdn = 3).

The comparative analysis also showed that dentists who had a family member infected with COVID-19 (Mdn = 13) had a higher score on the questionnaire assessing the severity of insomnia (z = −2.529, *p* = 0.011) than dentists who did not have a family member infected with COVID-19 (Mdn =10).

A significant positive correlation was identified between the total score of AIS and the total score for the FCV-19S questionnaire (r = 0.42 **, *p* < 0.001), indicated by the correlation coefficient which proved a medium effect size. This means that the more the dentists were afraid of COVID-19, the more they would suffer from insomnia.

Our results showed that there was a positive correlation between the total score of AIS and variables which refer to some of the doctors’ fears. Thus, we identified that the more the dentists were afraid of the fact that the patients did not declare the truth about their health, the more their insomnia would be severe (r = 0.29 **, *p* = 0.007). The more the dentists were afraid of the fact that they might be infected by their co-workers (r = 0.33 **, *p* = 0.002) or while taking off their protective suit (r = 0.26 *, *p* = 0.016), the more severe their insomnia would be. A negative correlation was identified between the total score of AIS and the fact that the patients respected COVID-19 preventive measures. We found that the more the patients respected these measures, the less the dentists suffered from insomnia (r =−0.26 *, *p* = 0.016).

For some dentists, wearing a protective suit became a difficult task because it prevented visibility and medical procedures. Thus, the more the dentists were concerned about the fact that wearing a protective suit would impede them from carrying out medical procedures *(r =* 0.40 **, *p* = 0.002) or about the fact that wearing a protective suit impeded visibility (r = 0.30 *, *p* = 0.020), the more they would have severe insomnia.

The statistical analysis proved that there were strong correlations between the anxiety state when dentists watched the news about the pandemic and both scores for fear and AIS. The results showed that the more anxious the dentists were when they followed news related to COVID-19, the higher both scores were (0.43 **, *p* ˂ 0.001 for AIS, and: r = 0.79 **, *p* ˂ 0.001 for FCV-19S). Positive correlations were also identified between AIS and other variables. Results are detailed in Table 4.

## 4. Discussion

Numerous studies conducted during the first year of the pandemic triggered by the Coronavirus infection have shown that there are vulnerable groups: women, people with minimal education, the unemployed, low-income individuals, and people living in the environment with high risk of infection [9,10,11,15]. Applied to the general population, the tool used to measure fear of Coronavirus infection scored highly, with most studies showing higher cutoff levels than the 16.5 score [16,22,23].

Dental offices had been closed for a long time, in terms of weeks, or months. Similarly, the number of patients had decreased considerably, with most patients showing that both patients and dentists preferred to intervene soon in case of urgent dental problems. However, although the financial impact had been significant, this was not crucial in increasing the fear of Coronavirus-19 infection. Similar results were highlighted by Gasparro et al. [35]. Researchers indicated that perceived job insecurity and fear of COVID-19 had negative impact on mental health, both variables being positively associated with depressive symptoms. The authors found that the effect of perceived job insecurity on depressive symptoms was weaker among those with a low fear of COVID-19. In a study conducted on Italian dentists, De Stefani et al. [36] also showed that more than half of the respondents declared that they were afraid because they were not sufficiently trained to restart work after the lockdown. They considered the virus infection highly dangerous, and they were concerned about the future economic situation of their clinical practices.

The scale proposed by Ahorsu et al. has been taken up by many researchers. Many published studies have validated the scale for use in various languages and in various countries [22,23,26]. The scale has also identified the level of fear of COVID-19 in populations in different countries. Some authors have argued that a weak point of the scale is that it did not propose cutoff scores, which made it impossible to make comparisons between populations, cultures, or ethnic groups [27,29,30,31]. Other research identified that different scores were highlighted by using the scale at various times during the first year after the onset of the pandemic. This showed that a high level was identified at the initial peak of the pandemic, after which the population became accustomed to the new preventive rules imposed to limit the spread of the virus. This peak in scores for fear of COVID-19 infection was also explained by the fact that little scientific information was available during the first months of the state of emergency.

The scale was applied to various populations of different countries but also on specific groups: students, doctors, medical professionals. The scale is useful for identifying the level of fear of infection and the results are useful for identifying people at risk (vulnerable populations, people from low socio-economic backgrounds, professions at risk of illness, patients with chronic diseases, etc.), but cannot be used for clinical purposes [16,18,22]. Some researchers have used the scale to provide a cutoff score, which is important when using a psychological tool. As Nikopoulou et al. [16] sustained, establishing a cutoff score is a common and useful practice in the psychiatry and psychology research fields to enable classification of respondents into either cases or non-cases. The authors proposed a cutoff score of 16.5 to separate both categories after having studied the Greek population.

In our study, the total score was 14.56 ± 6.90, lower than in other studies conducted on general healthcare professionals or dentists. Compared to other studies, the total score for FCV-19S was similar to those obtained by other investigations focusing on health professionals [27,28,29,30,31,32,33,34]. Firstly, this research was conducted on dentists and the respondents had more knowledge about infection, the spread of a virus and the implementation of protective practices. Secondly, this low score could be related to the period when the research was conducted, seven months from the beginning of pandemic. Compared to other studies, it is possible that the scores were lower compared to studies that measured the fear of COVID-19 during the peak of the pandemic [31,32,33,34]. Thirdly, the low score could also be related to easier access to medical supplies (a source of anxiety and fear during the first seven months of the pandemic). The fourth explanation could be related to the epidemiologic context, because in Romania the number of cases was not so high compared to other European countries (such as Italy, or Spain, where the number of cases and deaths increased considerably during the first seven months of pandemic).

Consolo et al. [7] identified similar results regarding the lower score compared to the general population. Their survey found that fear, anxiety, concern, sadness, and anger were commonly reported by dentists, but fortunately only a minority group reported a mild level of anxiety (10.3%), while 8.7% showed a score indicative of a severe level of anxiety. When thinking about COVID-19, only 4.2% of the questioned doctors reported an intense experience of fear. These results are similar to another survey conducted in Israel by Shacham et al. [9], in which elevated psychological distress was found in 11.5% of dentists. Similar to our study, anxiety was related to the level of fear of being infected by the patients. However, in our study we did not identify differences according to gender in terms of fear of contagion, these results being congruent with many others identified in the literature [21].

We found a positive correlation between the level of fear and the practice of some protective behaviors, results that were congruent with some studies conducted during the MERS or Ebola pandemics that proved the positive effect of fear on adopting preventive measures [37,38]. Therefore, normal fear of infection was associated with the adoption of recommended protection measures. Moreover, the implication of institutions, medical organizations, governments, and social entities was found to increase public compliance. Media, as well as social networks have the power to rapidly spread the information. When people have trust in medical institutions and policy makers, the power of fake news is lower, and compliance is higher [37]. In the case of the pandemic, the line between fear and anxiety will become less strong. Fear is related to a known or impending threat, whereas anxiety is mainly related to an imprecise or unknown threat [39]. So, the line will be created by the amount of knowledge that scientists, doctors, policy makers, media, or educators transmit about the virus. The higher the trust, the stronger the compliance. When these actors are missing, when they are undecided, when they do not transmit information or they transmit information which is contradictory, anxiety arise. The results of several studies indicated that public compliance with preventive behaviors like social distance, wearing masks, or vaccination during the health crisis required the development of social and institutional trust [40]. In our research, we found that breaking news about the pandemic had a negative impact on the mental state of dentists. We found a positive and strong relationship between the self-rated fear when dentists watched TV news about infection and scores for both fear of COVID-19 and insomnia. This result was due to the information related to the impact of the pandemic on health, financial incomes, food supplies or daily social activity. Trnka and Lorencova (2020) showed also that pessimistic communications used by the mass media considerably increasing traumatic feelings, fears and psychological distress in the Czech population during the outbreak of the pandemic [41] and Ermolaev et al. (2020) revealed that news about the speed of the spread of the coronavirus in Russia influenced people not to go to hospitals with minor health problems, when infection with the virus was seen to be putting their lives in danger [42].

Our study showed that dentists changed their preventive behaviors due to fear of infection. The results are congruent with other studies. For example, in a study conducted in thirty countries, Ahmed et al. (2020) showed that, despite the high level of medical knowledge among dentists, important levels of anxiety and fear were identified while working. The authors identified that a number of dental practices had been modified according to the recommended guidelines for emergency treatment and many offices had closed for an uncertain period [8].

The correlation between the fear of infection and the practice of preventive behaviors was also discussed by Becker et al. [43] in a study that included experts in dentistry from 32 countries. The authors identified preventive measures that were adopted by dentists in different countries. For example, 80% of the experts included in the research recommended wearing protective masks by patients even if they were not (at that time when the research was developed) recommended by professional associations or the WHO. These preventive behaviors could be explained by the fact that dentists had knowledge of and experience with contagious disease in dental settings. On the other hand, the high risks and fear of infection during maneuvers determined doctors to adopt supplementary strategies to diminish the risk of spread, even if they were not (yet) recommended.

We identified that dentists obtained a high score for insomnia (11.37 ± 3.45). As Nikopoulouet et al. [16] showed when they evaluated insomnia in the Greek population, results similar to those published by Sirajudeen et al. [25], an increasing amount of evidence indicated that there was a bidirectional relationship between psychosomatic conditions and insomnia and that this relationship may have been exacerbated by stress. In our study, the fear of infection and the anxiety related to financial matters during the first seven months from the outbreak of pandemic were related to the increased level of insomnia among our respondents. Our study showed that there was a strong positive correlation between total score of AIS and total score for FCV-19S questionnaire, meaning that the more the dentists were afraid of COVID-19, the more they would suffer from insomnia. Our results are similar to the few studies conducted on front-line medical staff working with COVID-infected patients. Xiao et al. [44] found that sleep quality was negatively associated with the degree of anxiety and stress and the effect of stress and anxiety on sleep was pointed out by many researchers [45,46] who also proved the negative impact on physical and mental health and the risk of developing chronic diseases.

Similar to our results, Bohlken et al. [47] showed that 18% of German doctors (psychiatrists and neurologists) reported that the pandemic triggered anxiety, while 9% reported sleep problems and Lai et al. [48] conducted a study on Chinese health care workers exposed to COVID-19 and proved that front-line health care workers had a high risk of developing unfavorable mental health outcomes and may have needed psychological support or interventions. Mustafa et al. [49] also identified fear of infection among dentists in South-Arabia, the main cause being contagion from patients. The researchers showed an increased concern about their accessibility to materials provided by dental health care authorities, which was mentioned as the best and safest approach for dealing with patients during and after the outbreak of pandemic.

We found that men where more prone than women to have sleep-related problems and that there was a strong positive relationship between both fear of infection and insomnia with self-rated fear when watching media news. The results of our study are in congruence with those obtained by Léger et al. (2020) who conducted a study on a French population. The researchers showed that media overexposure was associated with sleeping disorders during the lockdown and men were more prone to be overexposed to media and social media during lockdown [50]. Dai et al. (2020) identified that men had difficulties in maintaining sleep patterns and lifestyle during social distance restrictions and Sinha et al. (2020) showed that an increased digital media duration was evident in all age groups, mainly in males [51,52].

More studies should prove the effect of the pandemic on the physical and mental health of dentists Additionally, the long-term consequences of the fear of infection and of the application of measures to limit the disease need to be identified.

### 4.1. Reflections and Planning

Sleep quality is an indicator of physical and mental health. For healthcare professionals, sleep is an indicator of stress and burnout and these factors are the main cause of professional mistakes especially among doctors who are working in the frontline and under permanent pressure. Many studies have proven that sleep quality was closely related to addictive behaviors, burnout, malpractice complaints, poor communication with patients, low level of medical practice and suicide [53,54,55,56,57,58]. Due to the fact that fear of infection is an important cause of stress for dentists that is added to the overloaded activity during the pandemic period, dentists ’mental health must be an extremely important priority for hospitals and clinics. Psychological counselling, individual psychotherapy or Balint meetings must be provided to medical staff in order to help dentists to cope with the stress determined by the COVID-19 pandemic.

### 4.2. Strengths and Limitations of the Study

The most important strength of the study is that the results covered a great gap in scientific information related to the impact of fear of COVID-19 on mental health of medical professionals, especially of dentists. One of the limitations of the research is that the present study did not identify personality traits (according to the Big Five model, for example, which associated the presence of insomnia with neuroticism). Insomnia was more present in people with sleep vulnerability and introversion because all these were found to be linked to low gray matter density in the orbitofrontal cortex. Therefore, as Dekker et al. showed [59], introverts may be more vulnerable to COVID-19 fear and score higher on the insomnia scale.

Another limitation of the research is given by the small number of subjects. Thus, although the results are interesting, they cannot be generalized to the entire population of dentists, together with the geographical context related to the impact of the pandemic. So results cannot be generalized, and no causality is provided.

A third limitation of the research which should not be ignored is related to the fact that this study was conducted during the first part of the pandemic, when stress was connected to the fact that dentists did not have satisfactory access to protective materials or to complete information about the transmission of the virus, and were financially affected due to the closure of medical offices and the drastic decrease in financial profits. Therefore, sleep disorders could be strongly influenced by a combination of factors and not just by the fear of virus infection, and can affect quality of life [60].

The fourth limitation of the study is caused by the fact that the respondents were considered mentally healthy, no psychological or psychiatric diagnostic was obtained, and no supplementary psychological scale was applied. The presence of mental chronic diseases or various comorbidities could lead to a more pronounced presence of insomnia.

## 5. Conclusions

The COVID-19 pandemic had an important impact on the psychological health of dentists, especially during the first wave of lockdown. The fear of contagion, the closure of dental offices or the severe reduction of dental services, the collapse of financial incomes and the expensive preventive measures that started to be applied in dental settings with an important pressure on the budget, and also difficulties in terms of the impediment of visibility and of medical maneuvers had consequences on general well-being among dentists. The results of the studies must be interpreted considering the geographical context and the epidemiological particularities of each country during the fight against the COVID-19 pandemic. Dentists and policymakers must recognize that the COVID-19 pandemic has an impact on the physical and mental health of health professionals and dentists alike.

## Figures and Tables

**Table 1:** Sociodemographic and medical characteristics ^1^.

Sociodemographic and Medical Characteristics	*n* (%)/M ± SD
Age	37.81 ± 8.45
Length of employment (years)	11.87 ± 8.62
Number of working hours per week	30.94 ± 11.76
Gender	
Male	29 (34.94)
Female	53 (63.86)
I prefer not to say	1 (1.20)
Marital status	
Single	11 (13.25)
In relationship	72 (86.75)
Having children	
yes	46 (55.42)
1 child	22 (26.51)
2 children	19 (2.89)
3 children	4 (4.82)
4 children or more	1 (1.20)
none	37 (44.58)
Level of specialization	
Dentists	38 (45.78)
Male	17 (20.48)
Female	21 (25.30)
Residents	16 (19.28)
Male	1 (1.20)
Female	15 (10.64)
Specialists	10 (12.05)
Male	6 (7.23)
Female	4 (4.82)
Consultants	19 (22.89)
Male	5 (6.02)
Female	13 (15.66)
I prefer not to say	1 (1.20)
Teaching activity	
yes	23 (27.71)
no	60 (72.29)
Type of institution	
only public sector	3 (3.61)
only private sector	50 (60.24)
both private and public sectors	30 (36.14)
Working environment	
urban	77 (92.77)
rural	6 (7.23)
Having a chronic disease	
yes	9 (10.80)
no	74 (89.20)
Confirmed infected with COVID-19	
yes	3 (3.60)
no	80 (96.40)
Having at least one family member confirmed positive with COVID-19	
yes	21 (25.30)
no	62 (74.70)
Having periods during lockdown when living away from family	
yes	20 (24.10)
no	63 (75.90)

^1^ Number of answers (*n*) and percentage (%), Means and standard deviations (M ± SD).

**Table 2:** Fear of infection with COVID 19—self-rated items ^1^.

Items	M ± SD
I am afraid that wearing the protective suit against COVID-19 does not protect me enough	2.55 ± 1.41
I am afraid that the patients do not tell the truth about their health	3.47 ± 1.40
I am afraid I can get infected when I take off my protective suit	2.51 ± 1.43
I am afraid I might get infected from co-workers	2.54 ± 1.30
I am afraid of getting infected from my patients	2.78 ± 1.38
I believe that dentists have a very high risk of COVID-19 infection from their patients	3.52 ± 1.51
Dental procedures can be a source of infection and spread of COVID-19	2.81 ± 1.26
The fact that patients cannot wear a mask during the medical treatment causes fear of infection	2.48 ± 1.35
I try to shorten the duration of procedures for not staying in contact with patients too long	2.76 ± 1.51
I feel anxious when I watch the news and stories about COVID 19 on social platforms	2.49 ± 1.39

^1^ Means and standard deviations (M ± SD).

**Table 3:** Results for items of Athens Insomnia Scale (AIS) ^1^.

Items of the AIS	M ± SD
Sleep induction	1.40 ± 0.64
Awakenings during the night	1.55 ± 0.63
Final awakening earlier than desired	1.43 ± 0.49
Total sleep duration	1.47 ± 0.57
Overall quality of sleep	1.30 ± 0.55
Sense of well-being during the day	1.42 ± 0.64
Functioning (physically and mentally) during the day	1.29 ± 0.55
Sleepiness during the day	1.51 ± 0.59

^1^ Means and standard deviations (M ± SD).

**Table 4:** Correlation analysis between Fear of Covid-19 Scale, Athens Insomnia Scale and self-rated items.

Items	AIS	FCV19S
I am afraid that patients do not tell the truth about their health	r = 0.293 ** p = 0.007	r = 0.514 ** p = 0.000
I am afraid I can get infected when I take off my protective suit	r = 0.263 * p = 0.016	r = 0.635 ** p = 0.000
I am afraid I might get infected from colleagues	r = 0.334 ** p = 0.002	r = 0.603 ** p = 0.000
Wearing a protective suit impedes me from doing medical maneuvers	r = 0.409 ** p = 0.002	r = 0.142 *p* = 0.291
Wearing a protective suit impedes my visibility	r = 0.304 * p = 0.021	r = 0.210 *p* = 0.116
When I watch the news and stories about COVID-19 on social platforms, I feel anxious	r = 0.437 ** p = 0.000	r = 0.000 ** p = 0.799
Fear of COVID-19 Scale	r = 0.427 ** p = 0.000	1 -
Patients follow protective measures against COVID-19 infection	r = −0.265 * p = 0.016	r = −0.377 ** p = 0.000

* *p* < 0.05; ** *p* < 0.001.

## Data Availability

The data presented in this study are available on request from the corresponding author.
